# Decreased Spontaneous Eye Blink Rates in Chronic Cannabis Users: Evidence for Striatal Cannabinoid-Dopamine Interactions

**DOI:** 10.1371/journal.pone.0026662

**Published:** 2011-11-18

**Authors:** Mikael A. Kowal, Lorenza S. Colzato, Bernhard Hommel

**Affiliations:** Cognitive Psychology Unit and Leiden Institute for Brain and Cognition, Leiden University, Leiden, The Netherlands; University of Granada, Spain

## Abstract

Chronic cannabis use has been shown to block long-term depression of GABA-glutamate synapses in the striatum, which is likely to reduce the extent to which endogenous cannabinoids modulate GABA- and glutamate-related neuronal activity. The current study aimed at investigating the effect of this process on striatal dopamine levels by studying the spontaneous eye blink rate (EBR), a clinical marker of dopamine level in the striatum. 25 adult regular cannabis users and 25 non-user controls matched for age, gender, race, and IQ were compared. Results show a significant reduction in EBR in chronic users as compared to non-users, suggesting an indirect detrimental effect of chronic cannabis use on striatal dopaminergic functioning. Additionally, EBR correlated negatively with years of cannabis exposure, monthly peak cannabis consumption, and lifetime cannabis consumption, pointing to a relationship between the degree of impairment of striatal dopaminergic transmission and cannabis consumption history.

## Introduction

Cannabis (*Cannabis sativa*) is the most widely used illicit drug in Europe and the US. Its recreational use dates back to over 2000 years B.C. The active compounds in cannabis are called exogenous cannabinoids, with delta-9-tetrahydrocannabinol (THC) and cannabidiol (CBD) being responsible for most of the drug's psychoactive effects [1]. Current research indicates that THC, as a cannabinoid CB1 receptor agonist, indirectly affects dopaminergic functioning. Stimulation of the cannabinoid receptor type 1 (CB1) results in the release of dopamine (DA) [2]—a neurotransmitter involved in the control of goal-directed behavior, reward learning, reinforcement, and addiction [3]. However, CB1 receptors are not present at dopaminergic neurons. Instead, they are located in Gamma Amino Butyric Acid (GABA) and glutamatergic terminals which, in turn, influence DA/D1 and DA/D2 neurons by controlling DA inhibition. In other words, CB1 receptors contribute to the release of DA by inhibiting DA inhibitors.

Interestingly, the highest concentrations of CB1 receptors in the brain can be observed at the same areas where dopaminergic neurons are present [3]. Crucial regions in this regard seem to be the basal ganglia and, more specifically, the striatum, in which endogenous cannabinoids modulate the firing of DA neurons. This occurs through postsynaptic interactions between cannabinoids and DA at the level of G-protein/adenylyl cyclase signal transduction [4]. As a consequence, it makes sense to assume that any effect of THC on DA transmission is the product of an indirect process. This is different from the impact of other often abused drugs, like amphetamine or cocaine, which seems to act directly on DA neurons (for a discussion, see [5]).

Hitherto, two studies using Positron Emission Tomography have looked into the acute effect of THC on striatal DA transmission—with however inconsistent results: one study reported a THC-induced increase in striatal DA level [6] while another found no effect [7]. Things are even less clear with regard to chronic effects of long-term exposure to THC, on which no data are available. This is particularly unfortunate in view of Kuepper's et al. [8] suggestion that repeated THC administration may create a dopaminergic imbalance in the brain by increasing striatal DA levels but lowering DA levels in prefrontal cortex. As a possible consequence of this imbalance, chronic THC exposure has been assumed to induce psychotic symptoms in users [8]. However, a problem with this assumption is that it is not based on any evidence regarding chronic effects of THC on striatal DA transmission but on only one finding regarding the acute effects [6]. Therefore, it is not clear whether THC actually induces long-term dopaminergic imbalances.

To address this issue, the present study aimed at investigating the effect of long-term exposure to cannabis on striatal DA transmission. In the case of chronic effects, it is difficult to differentiate between the specific psychoactive plant components which caused the potential impairments. Consequently, we use the more generic term “cannabis” in the present study, even though the available data suggest that the observed effects are mainly due to the impact of THC. For one, from the two main studied psychoactive compounds of cannabis, only THC acts as a CB1 receptor agonist, while CBD functions as an antagonist. For another, CBD is suspected to reduce the psychotic effects of THC, which would suggest a role of CBD in diminishing the potential DA-impairing effects of THC [9]. Nevertheless, for the sake of precision, no reference to specific cannabinoids is made.

We assessed dopaminergic functioning by means of spontaneous eye blink rates (EBR), a well-established clinical marker of striatal DA production [10]–[12]. Numerous observations have helped to validate EBR as a measure of striatal DA functioning. For instance, deviant levels of EBR have been reported from patients suffering from DA-related impairments: While EBR is elevated in schizophrenic patients, who exhibit increased striatal DA transmission [13], EBR is lowered in Parkinson's patients, who have a reduced amount of nigrostriatal dopaminergic neurons [14]. In addition, EBRs vary as a function of the DRD4/7 genotype, which is associated with the modulation of DA level in the striatum [15]. Moreover, nonhuman primate research has shown that direct DA agonists and antagonists increase and decrease EBRs, respectively [16].

Exact predictions of how chronic cannabis use might affect the striatal DA level—and the associated EBR—can be derived from animal research. Hoffman et al. [17] showed that, in rats, chronic treatment with a CB1 receptor agonist results in a reduced sensitivity of CB1 receptors located at glutamatergic and GABAergic terminals. Moreover, chronic application of THC completely blocks long-term depression (LTD) of GABA-glutamate synapses in the striatum. Normally, the regulatory role of LTD is to inhibit the activity of GABA and glutamate neurons and, thus, to block their control over DA neurons, which again allows for DA transmission. Consequently, blocking LTD should reduce the extent to which endogenous cannabinoids modulate GABA and glutamate neuron activity. Moreover, the LTD-DA relationship appears to be bidirectional: striatal DA neurons are capable of synthesizing endogenous cannabinoids, which induce LTD and interact with DA as a supplementary inhibitory feedback mechanism [3], [4]. However, in the case of chronic cannabis use, the decreased sensitivity of CB1 receptors implies that the likelihood of endogenous cannabinoids evoking LTD is lowered. As a result of this bidirectional process, chronic application of exogenous cannabinoids present in cannabis could be expected to lead to decreased DA transmission due to long-term, maladaptive inhibition by GABA and glutamate [17]. If so, we would expect a decrease of spontaneous EBR in chronic cannabis users as compared to non-users.

## Results

EBR per minute was significantly lower in chronic cannabis users (M = 10.24; SD = 5.861) than in the non-user controls (M = 17.52; SD = 9.019), *t*(48) = 3.384, *p*<.01. The same effect was obtained in an ANOVA with group (chronic cannabis users vs. non-user controls) as independent variable and IQ and cigarette use as covariates: while the group effect was again significant, *F*(1, 46) = 5.477, *p*<.05, the covariate effects were not.

**Figure 1:**
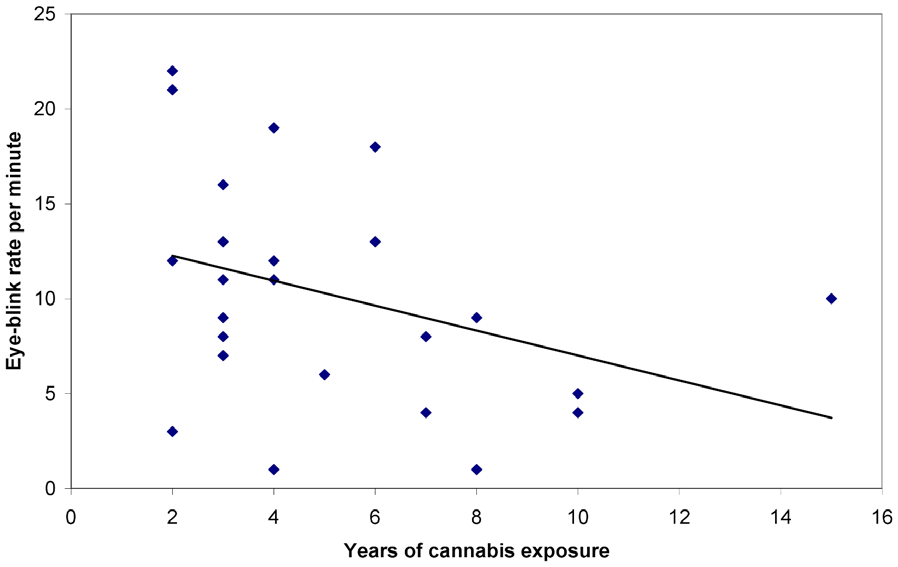
Years of cannabis exposure as a function of spontaneous eye blink rate per minute.

**Figure 2:**
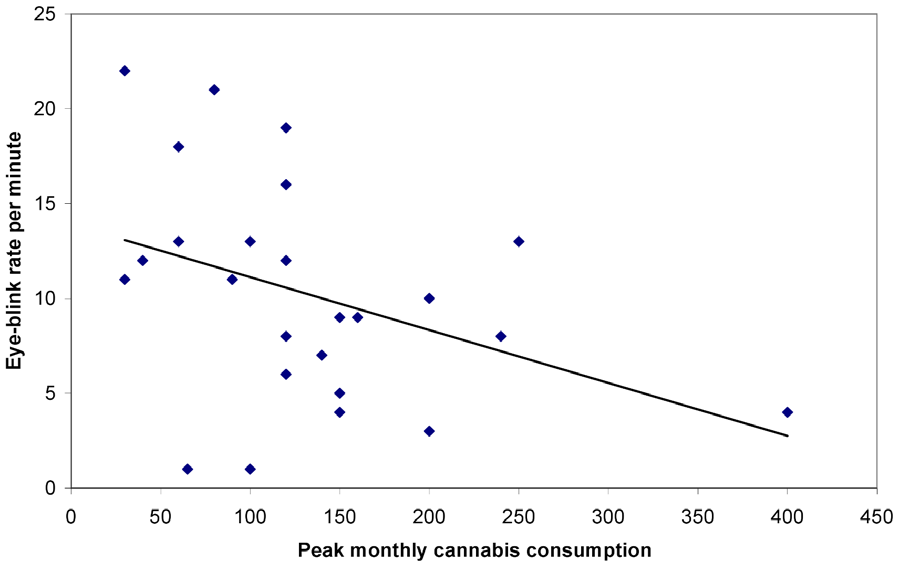
Peak monthly cannabis consumption (in joints) as a function of spontaneous eye blink rate per minute.

**Figure 3:**
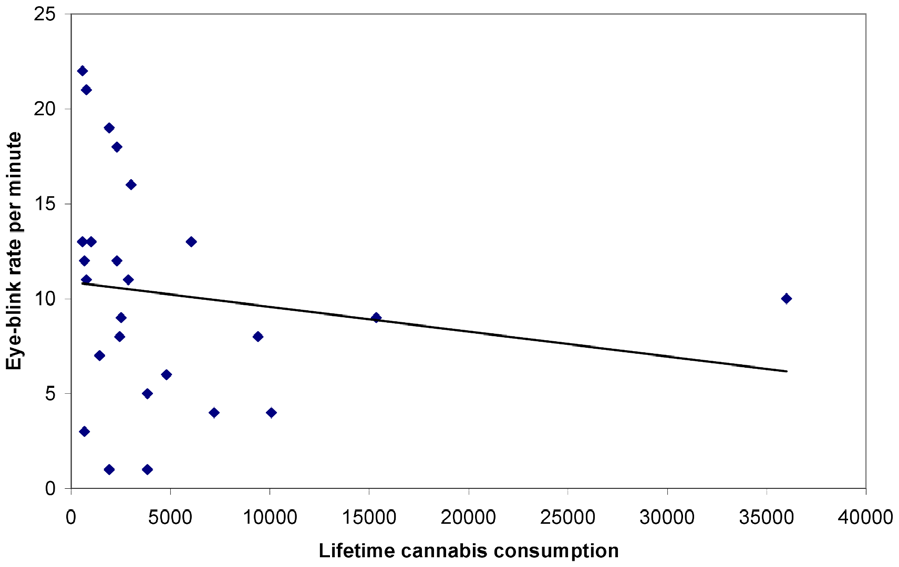
Lifetime cannabis consumption (in joints) as a function of spontaneous eye blink rate per minute.

To test whether the EBR in the chronic cannabis users was related to their consumption history and habits, Spearman's Rho correlation coefficients were calculated between EBR/minute and the years of cannabis exposure, age of onset, monthly regular, monthly peak and lifetime cannabis consumption. EBR correlated negatively with years of exposure, *r*(25) = −.42, *p*<.05 (see Figure 1), monthly peak consumption, *r*(25) = −.43, *p*<.05 (see Figure 2), and lifetime consumption, *r*(25) = −.40, *p*<.05 (see Figure 3), while no significant correlations were found for age of onset, *r*(25) = −.04, *p* =  n.s., and monthly regular consumption, *r*(25)  = −.25, *p* =  n.s.

## Discussion

The results of the study show a significant reduction of spontaneous EBR in chronic cannabis users, as compared to non-user controls. This can be interpreted as an indication of a dopaminergic hypoactive state in the striatum [10]–[12]. Additionally, a moderate negative correlation between EBR and years of cannabis exposure suggests that the degree of impairment of DA transmission is, to a certain extent, proportional to the period of cannabis use. Conversely, the lack of a correlation between EBR and the age of onset of cannabis consumption suggests that starting to use marijuana at an earlier age does not contribute to the level of dopaminergic hypoactivity. However, such a claim should be treated with caution due to the fact that adolescent cannabis use has been linked to specific cognitive impairments, like less efficient discrimination between relevant and irrelevant stimuli [18]. In any case, it can be assumed that the striatal dopaminergic hypoactive state of chronic cannabis users is the result of blocking the supplementary inhibitory mechanism of LTD. The impairment of GABA and glutamate neuron activity combined with the down-regulation of CB1 receptors seem to be plausible explanations for the observed decreased EBR in chronic users [3], [4], [17].

In the case of the modest negative correlation between EBR and monthly peak cannabis use, it could be inferred that a more pronounced binge use of marijuana has an additional detrimental impact on the level of DA in the striatum. However, DA impairment was found not to be related to the regular amount of cannabis consumed per month. A possible explanation for this effect comes from the research by Bolla et al. [19], who identified organic drug exposure intensity, instead of duration, as a key factor in developing drug-related neurocognitive deterioration. Therefore, it seems plausible to assume that binge use of cannabis is a better predictor of DA impairment than regular consumption. Additionally, the moderate negative correlation between EBR and lifetime cannabis consumption suggests that the degree of impairment of striatal dopaminergic functioning is related to the total amount of cannabis consumed during lifetime. Possibly, use of higher doses of cannabis, both in the short- and long-term, has a more detrimental enduring effect on GABA and glutamate inhibition of DA in striatum, as compared to the impact of using smaller doses for a longer period of time.

As for limitations of the present study, one is the lack of additional verification of participants' compliance with the no-consumption instructions. Subjects' urinary or plasma levels of THC metabolites (THC-COOH) were not examined to confirm cannabis use status. Another limitation is the correlative nature of the study, which does not preclude causal contributions from possible self-selection factors, such as a predisposition for low striatal DA production that seduces people to use cannabis. It may also be suspected that significantly more nicotine smokers in the chronic cannabis condition might have contributed to the difference in the observed EBR between groups. However, not only did the critical effect survive the input of nicotine use as covariate but research also indicates that the long-term effect of nicotine on DA is facilitatory rather than inhibitory [20]. This suggests that, in anything, the observed reduction in EBR provides a rather conservative estimate of the association between cannabis use and striatal DA levels.

Concluding, the results of the present study point to less efficient striatal dopaminergic functioning in chronic cannabis users. This finding seems crucial in understanding the suspected psychotic effects of long-term cannabis use and throws some doubts on the claim that cannabis-induced psychosis results from the combination of increased striatal and reduced prefrontal DA levels [8]. Additionally, the fact that cannabis has an indirect effect on DA implies caution in predictions of DA-related disorders due to chronic cannabis use. As a result of dopaminergic neurons not being impaired by cannabinoids, long-term consequences of cannabis exposure may be less severe than in the case of drugs directly damaging dopaminergic cells, like cocaine (for a discussion, see [5]). More research is required in order to identify the neurophysiological and cognitive effects of continuous marijuana use, which are likely to be more subtle than in the case of other recreational drugs.

## Materials and Methods

### Participants

Fifty-three healthy adults served as participants, 28 chronic cannabis users and 25 non-user controls. Participants received either course credit or financial reward. The sample was obtained from the city of Leiden using local advertisement, posts on community bulletin boards, and leaflets distributed in Leiden “coffee shops” (in which Dutch law permits selling/serving soft drugs to customers). Subjects were informed that they will participate in a study on the cognitive and neural effects of cannabis.

Following Colzato and Hommel [21], the inclusion criterion for cannabis users was a weekly consumption of at least 4 joints for a minimum of 2 years. The exclusion criteria were: (1) current or previous regular use of other drugs except for cannabis (regular use defined as having used a drug more than 3 times in a lifetime), (2) abuse of alcohol (more than 14 units per week), (3) history or presence of an Axis 1 psychiatric disorder (DSM-IV; assessed with the use of the Mini International Neuropsychiatric Interview; M.I.N.I. [22]), (4) clinically significant medical disease, and (5) use of psychotropic medication. Non-user controls were required to meet the same criteria, with the exception that they could not report current or previous cannabis use. Additionally, participants were not permitted to consume caffeine, chocolate, or alcohol 12 hours before the experimental session, or to use nicotine 2 hours before the study. It was also not allowed to use cannabis on the day of study. However, cannabis use on the previous day was accepted in order to minimize the impact of possible withdrawal effects of addicted chronic users. Within the study sample, two participants were rescheduled for another day due to non-compliance with the consumption avoidance requirements. Three individuals were excluded from the group of chronic users because of meeting the criteria for a psychiatric disorder.

Both groups were matched for age, gender, race (92% Caucasian, 8% Turkish), and IQ (measured by Raven's Standard Progressive Matrices; SPM [23]). The demographic and cannabis use statistics are presented in Tables 1 and 2, respectively. Additionally, in Table 1 the results of t-tests are presented to provide a comparison of demographic group characteristics. Written informed consent was acquired from all participants after they had been explained the nature of the study. The protocol and compensation for participants were approved by the institutional review board (Leiden University, Institute for Psychological Research).

**Table 1:** Demographic data.

	Non-user controls	Chronic cannabis users	Significance level
N (M:F)	25 (13∶12)	25 (19∶6)	n.s.
Age (years)	21.7 (3.8)	23.9 (4.4)	n.s.
Race	23 C : 2 T	23 C : 2 T	n.s.
Raven IQ	124.4 (5.6)	124.2 (7.6)	n.s.
Alcohol use	3.1 (2.4)	3.9 (2.8)	n.s.
Nicotine use	4 S : 21 NS	21 S : 4 NS	**

Standard deviation in parentheses; n.s.: non-significant difference; Race: C – Caucasian, T – Turkish; Raven IQ: measured by Raven's Standard Progressive Matrices; Alcohol use: consumption of units per week; Nicotine use: S – smoker, NS – non-smoker.

**p<.01.

**Table 2:** Self-reported cannabis use.

Sample	Mean (SD)
Years of exposure	5.4 (4.4)
Age of onset	18.4 (2.9)
Monthly regular use	62.5 (45.7)
Peak use in a month	131.8 (81.6)
Lifetime consumption	4895 (7409.4)

Standard deviation in parentheses; Monthly regular, monthly peak cannabis use and lifetime consumption: consumption of joints.

### Procedure and Design

Spontaneous EBR was recorded using a BioSemi ActiveTwo system (BioSemi Inc., Amsterdam, the Netherlands). The recording took place with two horizontal (one left, one right) and two vertical (one upper, one lower of right eye) Ag-AgCl electrodes. A vertical electrooculogram (EOG), which records the voltage difference between two electrodes placed above and below the left eye, was used to detect eye blinks. A horizontal EOG, which records the voltage difference between electrodes placed lateral to the external canthi, was used to measure horizontal eye movements in order to provide an online prevention of movement artifacts in the data. The EOG signals were digitized at 512 Hz. Data analysis was performed using Brain Vision Analyzer (Brain Products™ GmbH, Munich, Germany; http://www.brainproducts.com/products/analyzer/index_analyzer.html) with a high-pass filter of 1 Hz applied offline. Eye blinks were semi-automatically detected using the built-in Gratton and Coles [24] algorithm. Recordings did not take place after 5 p.m. due to spontaneous EBR being stable during daytime, but increasing in the evening (around 8:30 p.m. [25]). Participants were comfortably sitting in front of a blank poster with a cross in the center, located about 1 m from the subject. Participants were alone in the room and asked to look at the cross in a relaxed state. The recording lasted 6 minutes. Individual EBR was calculated by dividing the total number of eye blinks during the 6-min measurement interval by six.
